# Difficult tracheal intubation in critically ill

**DOI:** 10.1186/s40560-018-0318-4

**Published:** 2018-08-13

**Authors:** Armin Ahmed, Afzal Azim

**Affiliations:** 1 Department of Critical Care Medicine, King George Medical University, Lucknow, 226003 India; 2 Department of Critical Care Medicine, Sanjay Gandhi Postgraduate Institute of Medical Sciences, Lucknow, 226014 India

**Keywords:** Critically ill, Intubation, Physiologically difficult airway

## Abstract

**Background:**

Endotracheal intubation in critically ill is a high-risk procedure requiring significant expertise in airway handling as well as understanding of pathophysiology of the disease process.

**Main body:**

Critically ill patients are prone for hypotension and hypoxemia in the immediate post-intubation phase due to blunting of compensatory sympathetic response. Preoxygenation without NIV is frequently suboptimal, as alveolar flooding cause loss of alveolar capillary interface in many of these patients. All these factors, along with relative fluid deficit, neuromuscular fatigue and coexistent organ dysfunction lead to physiologically difficult airway. Airway in ICU can be classified as anatomically difficult, physiologically difficult and anatomically as well as physiologically difficult. Though rapid sequence intubation is the recommended method for securing airway in these patients, other methods like delayed sequence intubation awake intubation and double setup approach can be used in specific subgroups. Further research is needed in this field to set guidelines and fine tune airway management for patients with specific organ failure or dysfunction.

**Conclusion:**

Airway in ICU should be managed according to the physiological as well as the anatomical abnormalities.

## Background

It is well known that intubation in critically ill is a high-risk procedure and different from operating room for various reasons like hemodynamic instability, hypoxemia, metabolic acidosis, raised intracranial pressure, and coagulopathy. Airway-related events in ICU are potentially fatal, thus giving minimal margin of error to the intensivist [1, 2]. Fourth national audit project of the royal college of anaesthetists and the difficult airway society reported that 61% of airway-related events in ICU were associated with death or permanent neurological damage compared with 14% in operating room [3].

Apart from patient factors, there are several factors including the environment of the ICU and the skill of the operators and assistants in the ICU that are different from operation theatre [4, 5].

The term anatomically difficult airway is used when there is difficulty in bag mask ventilation or difficulty in inserting supraglottic airway device or difficulty in visualisation of glottic opening or passing of endotracheal tube through the opening. Physiologically difficult airway is the one where the process of induction and intubation can be potentially life threatening due to reduced physiological reserves pertaining to disease process [6]. It is important to differentiate between anatomically and physiologically difficult airway because awake intubation is the gold standard in predicted anatomically difficult airway while awake intubation can worsen already deranged physiology of a critically ill patient such as inadequate blunting of airway reflexes can cause rise in intracranial pressure or worsen cardiac ischemia in the predisposed individuals.

Moreover, baseline physiological derangements worsen with increased number of attempts to intubate [7]. Therefore, airway strategy with highest rate of first pass success is important in management of these patients. There can be another group of patients who have anatomically as well as physiologically difficult airway (see Table 1). One can intuitively understand that a common approach of induction and intubation cannot be used for all types of conditions and specific subgroups require specific strategy modification. Current article deals with key components of airway management in critically ill patients and also address the finer details of physiologically difficult airway.

**Table 1:** Classification of airway in ICU

Anatomically difficult airway	Physiologically difficult airway	Anatomically as well as physiologically difficult airway
A. Difficult bag mask ventilation	1. Neurophysiologic derangement (raised intracranial pressure)	• A + (any of physiological derangement 1....8)
B. Difficult supraglottic device placement	2. Cardiovascular derangement (derangements of preload, afterload, contractility or rhythm)	• B + (any of physiological derangement 1....8)
C. Difficult intubation	3. Respiratory derangement (hypoxemia and hypercarbia)	• C + (any of physiological derangement 1....8)
D. Difficult surgical airway	4. Hepatic derangement (raised intracranial pressure and coagulopathy)	• D + (any of physiological derangement 1....8)
	5. Renal derangement (encephalopathy, pulmonary oedema, hyperkalemia and metabolic acidosis)	
	6. Gut derangement (raised intra-abdominal pressure, abdominal compartment syndrome)	
	7. Severe sepsis (lactic acidosis, distributive shock multiple organ dysfunction)	

## Basics of airway management in ICU

### Prediction of difficult airway in ICU

In order to address the prediction of difficult airway in critically ill patients, De Jong et al. developed and validated a score (MACOCHA) in a multicentre study including 1000 intubations in 42 ICUs [8].The score included seven parameters, out of which five were patient related (Mallampati >III or IV, obstructive sleep apnoea (OSA), reduced C-spine mobility, mouth opening < 3 cm), two parameters pathology related (saturation less than 80% and coma) and one parameter operator related (presence of non-anaesthesiologist). Each parameter has been given one point except for Mallampati and OSA which have 5 points and 2 points each respectively. The difficulty of intubation increases as the score increases from 0 to 12.

*Preoxygenation* is the process of creating an oxygen reservoir inside the lung which can be used during the apnoea time [9].This reservoir is created by removing nitrogen present in functional residual capacity (FRC) and replacing it with oxygen. Larger is the FRC, larger will be the reservoir and longer is the time to desaturate during apnoea. Rapidity of desaturation during apnoea is also affected by metabolic rate. At higher metabolic rate, oxygen will be removed rapidly from this reservoir and vice versa.

Oxygen consumption in an anaesthetised patient is around 250 ml/min. Amount of oxygen in lungs during normal tidal breathing is roughly 13% (290 ml; i.e. 13% of FRC). For an adult breathing at room air and normal oxygen consumption, the oxygen content of the lung will be consumed in 1 min after apnoea. Complete de-nitrogenation of the lung will give an oxygen reservoir equivalent to FRC, i.e. around 2000 to 2500 ml [10, 11].With oxygen consumption at the rate of 250 ml/min, this will be sufficient for up to 8 min. This reservoir is much smaller in critically ill (especially obese) patients due to reduced FRC.

Pre-oxygenation is generally done for 3 to 5 min in operating room to de-nitrogenate the lungs. Effectiveness of preoxygenation can be monitored by measuring fraction of oxygen in the expired gas (FeO2), which is considered a surrogate marker of alveolar oxygen content. There is limited data on duration of preoxygenation for adequate de-nitrogenation in critically ill patients. Extending duration of preoxygenation from 4 to 8 min has been shown to be only marginally effective or even harmful in one study on critically ill patients [12]. Current guidelines recommend 3 min of preoxygenation in critically ill patients, which should be done if possible by using non-invasive positive pressure ventilation (inspiratory pressure 5 to 15 cm, PEEP 5 cm and target tidal volume 6 to 8 ml/kg) in a head up position or with high-flow nasal cannula with oxygen flow at 70 l per min [4].

In spite of adequate de-nitrogenation, critically ill patients desaturate more rapidly due to *smaller FRC*, *higher rate of oxygen consumption* and *extensive alveolar flooding* (as seen in ARDS or cardiogenic pulmonary oedema). Alveolar flooding reduces the alveolar capillary interface available for gas exchange. Therefore, even if the oxygen reservoir is there in the lungs, passage of oxygen from the reservoir into circulation is hampered. These patients have increased alveolar arterial (A-a) gradient and should be pre-oxygenated using non-invasive positive pressure ventilation (NIPPV), which decreases shunt fraction by recruitment of collapsed alveoli [13].

*Apnoeic oxygenation/peroxygenation* is the oxygen given from induction of apnoea till beginning of positive pressure ventilation. Apnoeic oxygenation relies on mass flow of oxygen across the pressure gradient created between upper airway and alveoli due to constant uptake of oxygen by capillaries. Most essential criteria for successful apnoeic oxygenation are maintenance of patent airway while delivering oxygen during attempts to intubate. Apnoeic oxygenation is delivered via high-flow nasal cannula (HFNC) capable of delivering warm humidified oxygen at 40 to 60 l/min. In the absence of HFNC, standard nasal cannula at 15 l/min can be used after adequate de-nitrogenation. Complications of prolonged apnoeic oxygenation include hypercarbia, acidosis, hyperkalemia, raised intracranial pressure and pulmonary hypertension [11, 14].

A systematic review and meta-analysis on respiratory support during intubation in critically ill patients showed apnoeic oxygenation (4 RCTs, 358 patients) was associated with higher minimum SpO_2_ as compared to those who did not receive apnoeic oxygenation [15]. Apnoeic oxygenation was delivered via high-flow nasal cannula in 3 RCTs and via standard nasal cannula in 1 RCT. Another meta-analysis reported increased first pass success rate of intubation (probably due to more time available for intubation without desaturation), higher peri-intubation oxygen saturation and decreased rates of hypoxemia with apnoeic oxygenation [16].

Jaber et al. combined NIV for pre-oxygenation and HFNC for apnoeic oxygenation during intubation of hypoxemic patients in a RCT including 24 patients in the control group and 25 patients in the intervention group. Lowest SpO_2_ values were significantly higher in the intervention group [17].

It is clear from the above discussion that pre-oxygenation and apnoeic oxygenation are two key strategies for increasing safety of intubation in critically ill. It should be remembered that in patients with severe hypoxemia, these strategies might not work due to widespread damage to alveolar-capillary interface. Patients not maintaining saturation on NIV form a subgroup where pre-oxygenation and apnoeic oxygenation are likely to be ineffective. Therefore, prolonged NIV trials in severely hypoxemic patients are discouraged and these patients should be timely intubated using an ICU intubation care bundle. In their landmark paper, Jaber et al. showed a ten component-based intubation bundle approach was associated with significant reduction in both life threatening as well as other complications in ICU intubations [18]. Their intubation bundle included pre-oxygenation with non-invasive positive pressure ventilation, rapid sequence induction, two operators, cricoids pressure, capnography, fluids, sedation and vasopressors as per need.

*Capnography* is the continuous monitoring of partial pressure of carbon dioxide in the exhaled gases. The capnography waveform is achieved by plotting expired partial pressure of CO_2_ on the *Y* axis and time on the *X* axis. Methods of capnography measurement include side stream analyser and mainstream analyser. Capnography is widely used for various indications ranging from confirmation of tracheal intubation, monitoring adequacy of ventilation, estimation of cardiac output and monitoring of adequacy of cardiopulmonary resuscitation. More than 70% of ICU-related airway deaths in NAP4 study were associated with failure to use capnography in patients dependent on artificial airway [3]. Therefore, current guidelines recommend use of continuous capnography during intubation and tracheostomy for confirmation of correct placement of endotracheal or tracheostomy tube as well as for monitoring in all anaesthetised patients and patients requiring life support irrespective of their location [4, 5].

*Videolaryngoscopes* are used for management of difficult airway in operation theatre, intensive care units and emergency, as a rescue technique when direct laryngoscopy fails. These devices consist of fiberoptic indirect rigid laryngoscope with video camera mounted at one end. Intubation is done based on the video image of the laryngeal inlet which is magnified and requires less amount of suspension pressure to achieve adequate laryngeal view. Videolaryngoscopes are available in standard Macintosh blade design and acute angled blade designs. The acute angled blade design requires less neck manipulation, but stylet is needed for facilitation of tube insertion. A recently published systematic review including 64 randomised controlled trials on use of videolaryngoscopes vs. direct laryngoscopy for intubation in adults found reduced failed intubations with videolaryngoscopes especially while managing difficult airway [19]. Differential performance was reported with different designs. Current evidence does not show use of videolaryngoscope effects number of intubation attempts, duration required for intubation or incidence of hypoxic complications.

## Mode of induction (Table 2)

### Rapid sequence induction and intubation (RSII)

It is the method of induction in patients who are full stomach and at increased risk of vomiting and aspiration. RSII is used to minimise the duration between loss of airway reflexes and establishment of definitive airway with cuff inflation. RSII is the induction of choice in ICU patients because even if they are nil oral for significant duration, changes in electrolytes and metabolic milieu can lead to diminished gut motility and increased risk of aspiration. It is preferable to stop enteral feeding and remove gastric contents by gentle suction whenever possible before RSII.

**Table 2:** Modes of induction

	Salient features	Advantages	Disadvantages
Rapid sequence induction and intubation (RSII)	• Use of rapidly acting inducing agent (ketamine and etomidate preferred agents in critically ill) • Short acting muscle relaxant (succinylcholine or rocuronium) • Cricoid pressure	• Key strategy for patients at high risk for aspiration	• In inexperienced hands RSII can lead to CICO situation (cannot intubate, cannot oxygenate) • FONA (front of airway) techniques should be available as backup plan
Delayed sequence intubation (DSI	• Preoxygenation done after judicious use of sedation in delirious patients	• Key strategy in patients difficult to pre-oxygenate due to agitation	• Even low doses of sedation can cause blunting of airway reflexes and apnoea in critically ill patients
Awake intubation	• Intubation is done without use of muscle relaxant (spontaneous respiration is remains intact) • Flexiblescope intubation and videolaryngoscopy are used to aid awake intubation	• Key strategy in anatomically as well as physiologically difficult airway • Spontaneous respiration remains intact • Physiological compensatory response remains relatively intact	• Significant expertise and skill is required to perform awake intubation safely • Attempts to intubate without proper blunting of airway reflexes can precipitate laryngospasm and severe hypoxemia • Failed attempts can cause vomiting, aspiration, local injury and bleeding. • Critically ill patients can develop toxicity of local anaesthetics at very low doses due to compromised hepatic and renal function
Double setup approach	• Two approaches are prepared simultaneously in anticipated failed intubation.eg. RSII and surgical airway	• Increased safety and reduced time required for switching from one approach to other	• May increase the cost of care

RSII was first introduced by Stept and Safar in 1970 [20]. Since then, the technique gained popularity in emergency airway management of full stomach patients across the globe. Steps of rapid sequence induction include administration of inducing agent, use of muscle relaxant with rapid onset of action (succinylcholine or rocuronium) along with application of cricoid pressure. Current evidence shows significant variation and modification in RSII technique [21, 22]. Institutions should develop their own RSII protocols as per their requirements and patient population:

- Optimal positioning of the patient should be achieved (routinely sniffing position/ramp in obese/head up during preoxygenation to increase FRC/left lateral tilt in pregnant patients to avoid aortocaval compression by gravid uterus)
- Preoxygenation and per-oxygenation using non-invasive ventilation or high-flow nasal cannula
- Inducing agent (choice is dictated by hemodynamic status; ketamine preferred in hemodynamically unstable; dose of inducing agent can be pre-fixed or titrated as per response)
- Rapidly acting adjunct opioid to blunt laryngoscopy reflexes
- Application of cricoid pressure (10 N at the time of induction, which is increased to 30 N after loss of consciousness). Cricoid pressure should be removed if there is active vomiting, difficulty in laryngoscopy or passing of endotracheal tube. Supraglottic airway device placement requires removal of cricoid pressure. There is difference of opinion regarding the utility of cricoid pressure in preventing regurgitation. Current evidence neither favours nor refutes it use.
- Muscle relaxant (succinylcholine/rocuronium)
- Manual ventilation (though not a component of standard RSII, gentle manual ventilation can be used if oxygen saturation falls between induction and onset of paralysis). Risk of gastric insufflation during manual ventilation can be minimised by using proper positioning, gentle inflation pressure and cricoid pressure application.
- Maximising first pass (adjuncts bougie and stylets)
- Vortex approach (well-defined steps and swift transition from one plan to next in crisis situation without wasting time by performing multiple attempts with the same equipment and same technique). The vortex approach allows three attempts each with facemask ventilation, intubation and supraglottic device insertion. British guidelines also allow three attempts at intubation. However, there are data to suggest that more than two attempts at intubation outside the operation theatre are associated with high rate of complications.

### Delayed sequence induction (DSI)

It is the method of induction in patients who are agitated and difficult to pre-oxygenation [23, 24]. Small doses of ketamine or benzodiazepine can be used to calm the patient enough to tolerate pre-oxygenation. Dose titration is done in a way not to blunt airway reflexes or spontaneous respiration. Further doses of inducing agent are given after pre-oxygenation has been satisfactorily achieved. Ketamine can cause tachycardia and hypertension due to its sympathomimetic effect. Dexmedetomidine (alpha 2 antagonist) is an alternative in patients in patients who are tachycardiac or hypertensive. This method of procedural sedation has long been in use but the term delayed sequence intubation has been only recently coined by Weingart et al. in an observational study involving 62 patients requiring emergency airway management but difficult to pre-oxygenate due to altered mental status [25]. The study has received criticism for its design and sample size but DSI remains an important strategy in delirious patient population. DSI should be done by experienced anaesthetist as there are case reports of apnoea even with small doses of inducing agent.

### Awake intubation

It is the method of induction in which spontaneous respiration is preserved during securing the airway [26]. Awake intubation is the preferred method of predicted difficult airway management in operating rooms which are anatomically difficult airways. It has been advocated by some authors that “awake intubation” should be the method of securing airway in critically patients to prevent blunting of physiological compensatory response [27].

Topicalization of the airway, premedication with antisialogogues, H2 blocker and metoclopramide are the key components of awake intubation. Inadequate blunting of airway reflexes can precipitate laryngospasm, tachycardia, hypertension, etc. Flexible scope intubation, videolaryngoscopy, light wand, direct laryngoscopy, blind intubation, etc. are some of the methods for awake intubation. Significant expertise is required to perform awake intubation in critically ill patients safely.

### Double setup approach

It is a strategy of preparing for two approaches simultaneously in patients with anticipated failed intubation [28]. Cricothyroid membrane may be identified via ultrasound or clinically before inducing the patient for RSII. Similarly, preparation of surgical tracheostomy can be done before inducing a high-risk patient.

NAP 4 study reported an unexpected high failure rate of needle cricothyroidotomy. Failure to identify the cricothyroid membrane in stressful emergency situation was one of the causes [3]. It has also been found that when *cannot intubate cannot oxygenate* situation occurs, surgical airway is secured but mostly it is too late to prevent irreversible brain damage. Double setup approach increases the safety margin and helps overcome cognitive failure in emergency situation.

## Preparation for post induction loss of sympathetic drive and physiological compensation

ICU patients are under physiological stress due to their underlying disease. Many of them are in compensated state of their physiological derangement. Use of induction agents in these patients lead to “physiological compensation blunting or failure”. As a result, patient may undergo acute changes in hemodynamics and metabolic milieu at the time of induction and intubation [29]. Other factors contributing to the decompensation include lack of sleep, relative hypovolemia due to poor intake and increased work of breathing leading to diaphragmatic fatigue.

**Fig. 1:**
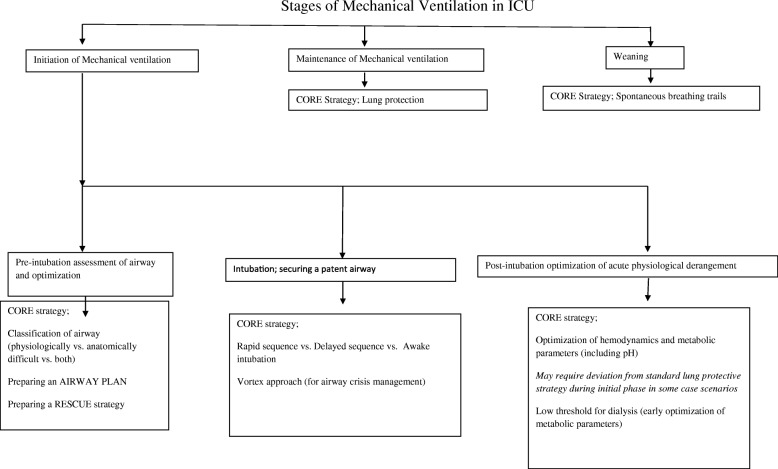
Stages of mechanical ventilation in ICU

Arterial line should be secured before induction and intubation of high-risk patients. If non-invasive blood pressure monitoring is being done, measurement cycle should be set at 1 to 2 min interval during induction and immediate post intubation period. Rapid changes in metabolic status should be monitored via regular blood gas analysis. It was shown that ICU-related airway problems occurred mainly in the post intubation phase. Understanding the underlying pathophysiology of particular disease process and preparing for post induction response of the patient can help alleviate such problems (e.g. fluid loading in hypovolemic patients, vasopressors infusion for hypotension, bicarbonate infusion for severe metabolic acidosis). Figure 1 describes the various stages of mechanical ventilation in critically ill patients.

## Difficult airway due to neurophysiologic derangement

Traumatic brain injury and stroke patients requiring intubation and mechanical ventilation form a specific subgroup where raised intracranial pressure is the key physiological derangement. These patients also show wide fluctuation of blood pressure during rapid sequence induction and intubation [30]. Table 3 shows the components of neuroprotection which authors use in their ICU while intubating such patients.

- Invasive blood pressure monitoring is preferable before induction as fluctuation in blood pressure can lead to increase in ICP and herniation. Fluid and electrolyte status should be evaluated meticulously before induction as these patients routinely get mannitol for decreasing cerebral oedema. Induction in volume depleted patients can lead to precipitous fall in blood pressure.
- Premedication with fentanyl/remifentanil is used to blunt the stress response due to laryngoscopy and associated increase in ICP. Use of lidocaine as an ICP control measure is weakly supported by evidence [31, 32]. Rapid bolus injection of lidocaine can cause hypotension, especially when used with propofol or thiopentone.
- Ketamine is no more considered contraindicated in head injury patients as newer prospective studies have not shown any association between increase intracranial pressure and ketamine use [33, 34]. Other inducing agents like propofol or thiopentone can cause significant drop in blood pressure and should be used with caution.
- Fasciculations caused by succinylcholine can cause rise in intracranial pressure. De-fasciculation dose with non-depolarising blocker should be given before its use.

**Table 3:** Neuroprotective strategy in ICU

• Head neutral	
• Head up	
• No compression on internal jugular veins	
• Maintain normal intrathoracic pressure, avoid airway obstruction, brochospasm, straining, coughing	
• Premedication before intubation with	
◦ Fentanyl 2 mcg/kg (caution for bradypnea, bradycardia and respiratory arrest)	
• Rapid sequence induction with ketamine and midazolam or ketamine and propofol or etomidate	
• Muscle relaxant	
◦ Rocuronium preferable	
◦ Succinylcholine (fasciculations can cause increase in ICP; hyperkalemia in patients with extrajunctional receptors)	
• Hemodynamic and metabolic targets	
➢Mean arterial pressure MAPs 90 to 100 mmHg (titrate according to baseline MAP± 20%)	
➢PaO2 80 to 100 mmHg	
➢PaCO2 30 to 35 mmHg	
➢pH 7.35 to 7.45	
➢Normoglycemia	
➢Normothermia	
➢Adequate sedation/analgesia	
• Potential cerebrovasodilators should not be used	
➢Vasopressin	
➢Calcium channel blockers	
➢NTG	
➢Nitroprusside	
• For acute rise in intracranial pressure	
➢Hyper ventilate to achieve PaCO_2_ 25 to 30 mmHg	
➢Hypertonic saline OR mannitol bolus	
➢Deepen sedation (boluses of midazolam)	

## Difficult airway due to cardiovascular derangement

Intubation in cardiac patients requires preload, afterload, heart rate and contractility optimization. Cardiac tamponade forms a specific subgroup where intubation should be delayed till definitive management of cardiac tamponade is achieved. Screening bedside ECHO is helpful in assessment of preload, afterload and contractility.

Management goals in patients at high risk for myocardial ischemia are avoiding increase in heart rate and factors that cause extreme increase in wall stress, i.e. inotropy and afterload [35]. Etomidate is the most cardio stable inducing agent but it does not blunt the stress of laryngoscopy [36].It can be combined with rapid onset opioids for intubation. Patients with left ventricular hypertrophy have poor diastolic compliance and depend on atrial kick for ventricular filling. These patients should be managed with strategy based on avoidance of atrial arrhythmias, hypotension and myocardial ischemia. Patients with regurgitant lesions (aortic/mitral regurgitation) are managed by maintaining slightly higher heart rate, low after load, adequate preload and support of contractility if impaired.

Patients with stenotic lesions are most challenging to manage. Hemodynamic goals in management of mitral stenosis include prevention of increase in pulmonary artery pressures, heart rate and marked afterload reduction. Aortic stenosis requires heart rate control, adequate preload and avoidance of myocardial ischemia.

## Difficult airway due to respiratory derangement

Pre-oxygenation with NIV is the most important component of airway management strategy in respiratory failure due to ARDS. These patients are prone for rapid recruitment thus putting lot of time pressure on the intensivist. Bag mask ventilation cannot be relied upon as a rescue strategy in these patients as severe ARDS lung is difficult to inflate with bag mask. Attempts to bag mask may cause unnecessary waste of time. Bag mask should be replaced with NIV. Apnoeic oxygenation as well as pre-oxygenation are important components of airway management in ARDS patients, but it should be noted that these are the very patients where pre-oxygenation and apnoeic oxygenation are likely to be least effective.

ARDS patients are prone of acute right ventricular dysfunction. If time permits screening, bedside ECHO should be done to evaluate right ventricle. Invasive hemodynamic monitoring is preferable at the time of induction in patients with concomitant cardiac and respiratory failure. Intubation in patients with right ventricular failure is extremely risky because unlike left ventricular function, right ventricular function deteriorates with increased intra-thoracic pressure caused by positive pressure ventilation. Right ventricular preload and afterload optimization is needed before intubation in order to prevent cardiovascular collapse. Methods of right ventricular afterload optimization include inhaled pulmonary vasodilators like nitric oxide or epoprostenol, correction of hypoxic pulmonary vasoconstriction by oxygen supplementation and decreasing atelectasis via non-invasive ventilator support. For further details, see reviews by Hrymak et al. and Krishnan et al. [37, 38].

## Difficult airway due to hepatic derangement

Hepatic failure patients have raised ICP due to hepatic encephalopathy. They should be intubated with neuroprotective strategy. Moreover, they have coagulopathy and are frequently thrombocytopenic. Suboptimal intubating conditions can lead airway trauma and bleeding, thus making physiologically difficult airway also anatomically difficult.

Nasal intubation should not be used in these patients in view bleeding tendency. Blood product transfusion should be considered in patients with high risk of bleeding.

## Difficult airway due to renal derangement

Renal failure patients may require intubation due to increased work of breathing caused by severe metabolic acidosis. These patients maintain their pH within normal range by increasing minute ventilation and washing out carbon dioxide. Induction of anaesthesia causes fall in minute ventilation and loss of compensatory response. Using neuromuscular blocking drug can further bring down the pH and cause precipitous fall in blood pressure and dangerous rise in potassium concentration. These patients should be intubated keeping rapid changes in metabolic milieu in mind.

- Succinylcholine causes hyperkalemia and should be avoided in patients with renal failure [39]. Rocuronium is the drug of choice while intubating this subgroup.
- Vasopressor should be connected via large bore cannula in the time of induction.
- Sodabicarbonate infusion can be considered in patients with pH < 7.2 before induction.

## Difficult airway due to gut dysfunction

ICU patients may suffer from paralytic ileus, ascites, pseudo-obstruction and raised intra-abdominal pressure. All these factors predispose for vomiting and aspiration till the airway is secured with cuff inflation. Rapid sequence induction is the preferred mode. Fluid shifts in patients in paralytic ileus, pancreatitis and intestinal obstruction predispose for hypotension during induction.

## Difficult airway due to sepsis

Sepsis patients are prone for distributive shock. Hemodynamic instability, lactic acidosis and coagulopathy are frequent issues. Volume resuscitation and vasopressor form the key components of airway management strategy. Optimal venous access should be secured before induction in these patients.

Invasive hemodynamic monitoring is preferable in patients requiring high-dose noradrenaline. Etomidate can cause cortisol insufficiency. Ketamine is the preferred agent for induction.

## Conclusion

- It is important to recognise that intubation in the ICU is different from the operating room. Intubation strategy in critically ill requires modification as per the physiological derangement.
- Airway in the ICU can be classified as anatomically difficult, physiologically difficult and anatomically as well as physiologically difficult.
- Though rapid sequence induction is the core strategy for induction of physiologically difficult airway, strategy modification like awake intubation with videolaryngoscope or flexiblescope intubation and delayed sequence intubation can be used by experts in certain high-risk subgroups.
- Preoxygenation and apnoeic oxygenation via NIV and high-flow nasal cannula are useful methods to increase safe apnoea time.
- Choice of inducing agent and muscle relaxant is dictated by patient’s pathophysiology.

## Abbreviations

A-a gradient: Alveolar arterial gradient
ARDS: Adult respiratory distress syndrome
DSI: Delayed sequence induction
FeO_2_: Fraction of oxygen in the expired gas
FRC: Functional residual capacity
HFNC: High-flow nasal cannula
ICP: Intracranial pressure
ICU: Intensive care unit
NAP4: National audit project 4
NIPPV: Non-invasive positive pressure ventilation
OSA: Obstructive sleep apnoea
PEEP: Positive end-expiratory pressure
RCT: Randomised controlled trial
RSII: Rapid sequence induction and intubation
